# Improved cell disruption of *Pichia pastoris* utilizing aminopropyl magnesium phyllosilicate (AMP) clay

**DOI:** 10.1007/s11274-013-1262-z

**Published:** 2013-01-30

**Authors:** Sun-Il Kim, Yuanzheng Wu, Ka-Lyun Kim, Geun-Joong Kim, Hyun-Jae Shin

**Affiliations:** 1 Department of Chemical and Biochemical Engineering, Chosun University, #375 Seosuk-dong, Dong-gu, Gwangju, 501-759 Republic of Korea; 2 Department of Biological Sciences, College of Natural Sciences, Chonnam National University, #300 Yongbong-dong, Buk-gu, Gwangju, 500-757 Republic of Korea

**Keywords:** AMP clay, Cell disruption, *Pichia pastoris*, Virus-like particles

## Abstract

An efficient method for *Pichia* cell disruption that employs an aminopropyl magnesium phyllosilicate (AMP) clay-assisted glass beads mill is presented. AMP clay is functionalized nanocomposite resembling the talc parent structure Si_8_Mg_6_O_20_(OH)_4_ that has been proven to permeate the bacterial membrane and cause cell lysis. The recombinant capsid protein of cowpea chlorotic mottle virus (CCMV) expressed in *Pichia pastoris* GS115 was used as demonstration system for their ability of self-assembly into icosahedral virus-like particles (VLPs). The total protein concentration reached 4.24 mg/ml after 4 min treatment by glass beads mill combined with 0.2 % AMP clay, which was 11.2 % higher compared to glass beads mill only and the time was half shortened. The stability of purified CCMV VLPs illustrated AMP clay had no influence on virus assembly process. Considering the tiny amount added and simple approach of AMP clay, it could be a reliable method for yeast cell disruption.

*Pichia pastoris* has become an efficient expression system for producing recombinant proteins of intracellular and extracellular origin from different sources at high yields (Cereghino and Cregg 2000). However, intracellular products expressed in *P. pastoris* require an additional step to disrupt the mechanically rigid cell wall, which is composed of multiple layers of cross-linked β-1,3-glucan, chitin and glycosylated mannoproteins (Kollár et al. 1997; Smits et al. 2001). Common protocols of cell wall disruption include breaking the cell wall either mechanically or enzymatically, which are usually tedious and time-consuming (Geciova et al. 2002; Stowers and Boczko 2007). The improvement of disruption methods is essential to acquire higher protein yields with low cost and good reproducibility.

In this work, we developed a simple method to disrupt *Pichia* cells utilizing glass beads mill combined with organic clay. Aminopropyl magnesium phyllosilicate (AMP) clay is a sandwiched organo-functionality with layered lamella sheets ranging from 20 to 200 nm that resemble talc parent structure Si_8_Mg_6_O_20_(OH)_4_ (Ferreira et al. 2008; Lee et al. 2010). It has been reported that AMP clay at high concentration displays antimicrobial activities against *Escherichia coli*, *Staphylococcus aureus* and *Candida albicans* by permeating the bacterial membrane and causing cell lysis (Chandrasekaran et al. 2011). Therefore, the applicability of AMP clay to enhance cell disruption was investigated to assess its efficiency on cell lysis. The demonstration system involves the recombinant capsid protein (CP) of cowpea chlorotic mottle virus (CCMV) which was expressed in *P. pastoris* GS115 and spontaneously assembled into icosahedral virus-like particles (VLPs) (Wenger et al. 2007; Wu et al. 2011).

The recombinant strain G48 was constructed by inserting CCMV CP into *Pichia* integrative vector pPICZ A under the highly-inducible AOX1 promoter (Wu et al. 2011). As described in EasySelect *Pichia* Expression Kit (Invitrogen, USA), recombinant G48 was inoculated in 25 ml buffered glycerol-complex medium (BMGY) at 30 °C with shaking at 240 rpm for 16–18 h and then transferred into 100 ml buffered methanol-complex medium (BMMY) for 72 h induction. *Pichia* cells were centrifuged at 4000×*g* for 10 min for disruption. The pellets were washed with distilled water and resuspended in breaking buffer (50 mM sodium phosphate, pH 7.4, 1 mM EDTA, 5 % (v/v) glycerol, and freshly made 1 mM PMSF). An equal volume of acid-washed glass beads (0.5 mm, Sigma) and 0.2 % (w/v) AMP clay; glass beads only; or 0.2 % AMP clay only were added to the cell suspensions. In a primary experiment the concentration of 0.2 % was chosen from a serial of 0.2, 0.5 and 1 % as the most suitable amount of AMP clay added. The mixture was agitated as follows: a 30 s vortex followed by an interval of 30 s on ice and sample collection after every 2 min. The samples were centrifuged at 4000×*g* for 10 min at 4 °C, and protein concentration of the supernatant was measured by Bradford assay. The experiments were repeated 5 times and each figure was measured 3 times for average. As shown in Table 1, the starting concentrations of all samples (from 0 to 2 min) were too low to show any difference; as time went on, the results of AMP clay-assisted glass beads and glass beads mill only method displayed statistically significant difference (*p* = 0.038). In the combined method, the protein concentration increased and reached the highest level at 4.24 mg/ml after 4 min. With the beads mill only method, the highest protein concentration obtained was 3.81 mg/ml at 8 min. The disruption time assisted with AMP clay was half shorten, while the protein concentration extracted increased by 11.2 %. As expected, the methods using the negative control or AMP clay only did not yield proteins at a detectable level. Similar results were also observed when AMP clay was combined with sonication.

**Table 1:** Total protein concentration of *P. pastoris* G48 by different cell disruption methods

Time (min)	Total protein concentration by AMP clay-assisted glass beads (mg/ml)	Total protein concentration by glass beads only (mg/ml)	Total protein concentration by AMP clay only (mg/ml)
0	0.07 ± 0.02	0.07 ± 0.03	0.08 ± 0.02
2	0.32 ± 0.06	0.39 ± 0.03	0.09 ± 0.04
4	4.24 ± 0.04 *	3.57 ± 0.05 *	0.09 ± 0.03
6	4.04 ± 0.05 *	3.59 ± 0.03 *	0.11 ± 0.05
8	3.85 ± 0.03 *	3.81 ± 0.04 *	0.11 ± 0.04
10	3.74 ± 0.03 *	3.58 ± 0.02 *	0.12 ± 0.03

* The difference in the mean values of the two groups is greater than would be expected by chance; there is a statistically significant difference between the input groups (*p* = 0.038)

The supernatant samples were diluted ten times and checked by SDS polyacrylamide gel electrophoresis (SDS-PAGE, Bio-Rad Mini-PROTEAN 4-gel electrophoresis cell, 15 % polyacrylamide gel, 120 V for 2.5 h). As presented in Fig. 1, there was a significant increase in protein extraction from 0 to 2 mg/ml at 4 min using both the AMP clay-assisted beads mill and the beads mill only; however, all the bands from the method using AMP clay only were obscure. It’s interesting to find that the amount of CCMV CP (approximate 20 kDa, boxed bands in Fig. 1) obtained from the combined method was much higher than that from the method using glass beads only: 0.82 mg/ml compared to 0.73 mg/ml (calculated by software BandScan v5.0). This indicated that AMP clay could preserve coat protein from degradation during disruption.

**Fig. 1:**
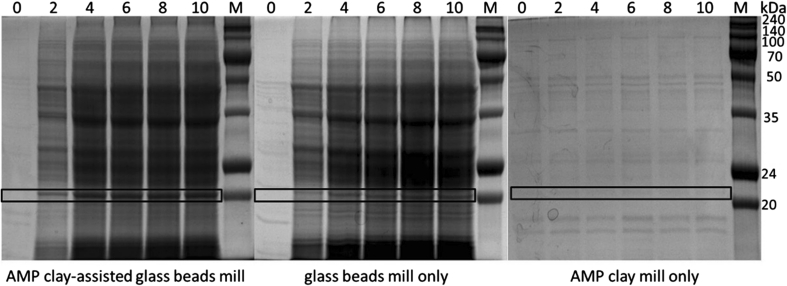
Comparison of SDS-PAGE results by different cell disruption methods. Time 0, 2, 4, 6, 8, 10 min; the boxed band region was recombinant CCMV capsid protein; M, DokDo-MARK™ Broad-range

To check the influence of AMP clay on the lysis process, oil-immersion light microscopy was introduced to assess cell breakage and debris size using Nikon Eclipse TS100 microscope (Nikon, Japan). Samples were diluted to the equivalent of a 1 % cell slurry solution and examined at 400× magnification. From Fig. 2, the lysate from the AMP clay-assisted method showed a change from the initial aggregation to small cellular debris with large and irregular in shape. This change may be attributed to the binding of *Pichia* cells with AMP clay in the solution via electrostatic interactions. In contrast, the lysate from the glass beads only method revealed extensive fragmentation of cells with almost no large particles of aggregated cellular debris visible after the disruption.

**Fig. 2:**
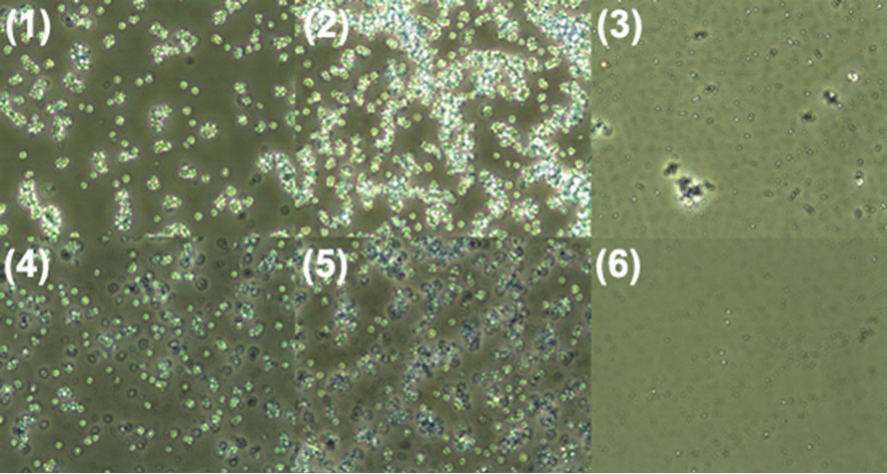
Microscopy pictures of *Pichia* cell disruption by different methods. (1), (2), (3) were from AMP clay-assisted glass beads mill as time of 0, 4, and 10 min; (4), (5), (6) were from glass beads mill only as time of 0, 4, and 10 min as control

Subsequently, a modified viral capsid purification procedure based on polyethylene glycol (PEG) precipitation and density gradient centrifugation was used to purify CCMV VLPs (Ali and Roossinck 2007). The concentration of purified CCMV CP by AMP clay assisted method was 0.28 mg/ml, which was a little higher compared to 0.23 mg/ml obtained by glass beads mill only. The recovery efficiency of target product by the combined method was 6.6 % of total protein (4.24 mg/ml) while it was 6.1 % of total protein (3.81 mg/ml) in the unmodified method. The purified CCMV VLPs were reassembled in sodium acetate buffer and analyzed by transmission electron microscopy (TEM) with High-Resolution Transmission Electron Microscope JEOL JEM 3010 (Electron Microscopy Laboratory, KAIST, Korea). CCMV CP reassembled into spherical VLPs with an average diameter of 28 nm as observed in Fig. 3. This result was identical to that of the control disrupted by the beads mill, implying that the assembly of viral capsids was not affected by AMP clay. Another recombinant GS115/pPICZ/lacZ (Mut^+^) carried a fused lacZ gene was also disrupted by both methods to check whether AMP clay would affect the functionality of recombinant proteins in *P. pastoris*. Similar enzyme activity of crude β-galactosidase was observed in both methods. This suggested that AMP clay caused no denaturation or side effect on enzyme activity of recombinant proteins in *P. pastoris*.

**Fig. 3:**
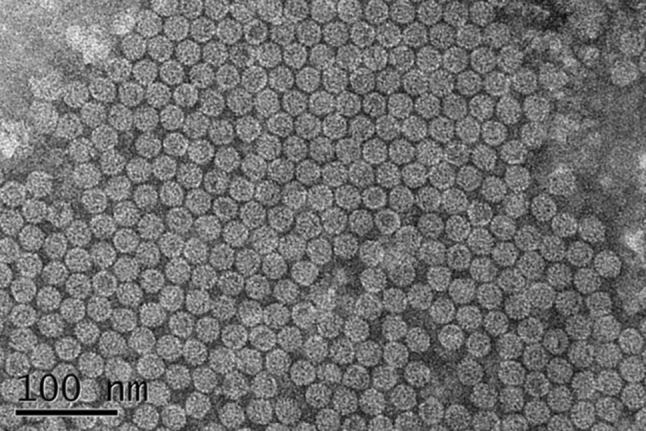
TEM picture of CCMV VLPs by AMP clay combined glass beads mill. The spherical particles showed an average diameter of 28 nm

As demonstrated by Lee et al. (2010), AMP clay was an organoclay-modified derivative in the form of nanocomposite Si_8_Mg_6_O_20_(OH)_4_. It was easy and inexpensive to synthesize and the amount added here was quite little which would not cause the increase of material cost. From the parent structure, AMP clay possesses hydrophilic and protonated groups (R-NH_2_) which create a number of binding sites for ion exchange within the interlayer spaces and serve as surface groups on the lamella (Holmström et al. 2007). The interaction between AMP clay and the negatively charged layer of glucan, chitin and mannoproteins destroyed the rigidity of cell wall, encouraged cell wall leakage and increased permeability, which ultimately lead to cell lysis. Under the high shear stress resulting from the collision between cells and glass beads in vigorous agitation, the addition of AMP clay may enhance the lysis process significantly. Considering the higher yield of CCMV CP and well-assembled VLPs, the spaces between the clay sheets may shelter the proteins extracted from the cytoplasm in the buffer and preserve them from degradation caused by glass beads.

In conclusion, a modified AMP clay-assisted glass beads mill was provided for *Pichia* cell disruption at the laboratory scale without cost increase. The treatment time was shortened by half, while the yield of extracted proteins was 11.2 % higher. The stability of CCMV VLPs demonstrated this method to be a simple and reliable method for yeast cell lysis.
